# Editorial Department

**Published:** 1854-04

**Authors:** 


### EDITORIAL DEPARTMENT.

(*Explanatory.*)

Mr. Editor:—In an editorial on the subject "Legalized Dissections" in your last issue, I find the following:

"In the government school at Ann Arbor, there has been an almost complete destitution. We are credibly informed that only one dissection class had been organized at the close of the third month of the term. The lecturer on anatomy had at that time no subject, and was, at the last accounts, waiting the arrival of one procured from a distant city, but which was delayed somewhere upon the route."

The facts are as follows: At the close of the third month of the term, *fifteen classes had been supplied*, and I had used *three* subjects in demonstrating my anatomical course, thus keeping fresh *material* constantly upon my lecture table.

As you were undoubtedly honest in the above statement, I trust you will do us the justice to give the same publicity to this correction, that you did to the error. MOSES GUNN.

(*Copy.*)

Buffalo, 27th Feb., 1854.

Dr. M. Green,

*Dear Sir:*—Dr. Daniels handed me your communication this morning. I take this, my first leisure, to reply.

The March No. of the Journal is already in type. The April number will contain your statement, followed by this explanation.

I was told, early in January, by the agent employed by your school to procure subjects in this city, that fifteen classes had at that time been formed, and only one of them supplied with material; that the professor of anatomy had no subject for his demonstrations; and that he had some time previously forwarded one to him, which was delayed somewhere upon the route. He gave, as his authority, Mr. ———, one of your students, who had made two unsuccessful trips hither in quest of material.

When he made this statement I objected, strongly, to his procuring material here, as if any discoveries took place the trouble would fall upon me.

Perhaps he deceived me, or perhaps was himself deceived. I have hitherto deemed him trustworthy, and do not know why he should tell anything but the truth.

You will see that my authority for the statement was sufficient. I must, however, give entire credence to your reply as will, no doubt, all who read it. Indeed, we have only to suppose a fortnight's difference in the time stated and both statements may be entirely correct. I do not wish you to look upon this explanation in the light of an apology. It is simply an act of justice, such as I shall always cheerfully render when any statement of mine is corrected. Neither would I have it understood that I do in any sense, withdraw from the positions I have assumed relative to the propriety of instituting government schools of medicine, or their efficiency when established.

With sentiments of the highest respect for yourself,

I remain,

Yours very truly,

SANFORD B. HUNT.

Prof. Moses Gunn.

The above correspondence will explain itself. We supposed that it was a sufficient apology for the statement made.

Three weeks after this correspondence passed, the Peninsular Journal of Medicine comes to us containing, as its leading article, a shower of abuse upon the Buffalo Journal, the Buffalo College, and more especially upon us the junior editor. In this article an anonymous writer, seconded and endorsed by the editor, impugns our veracity and our motives, descending to the lowest personalities, and applying the most opprobrious epithets.

To any such attack we can make but this reply. We are not competent to a war of billingsgate—we have not the necessary command of a peculiar dialect. We have repeatedly afforded to the Peninsular Journal an opportunity to refute, if possible, our arguments upon the main question at issue, viz., the propriety and usefulness of government schools. This issue we are willing to enter upon. We will cheerfully hold a controversy with the "Peninsular" upon it, if the discussion can be kept clear of personalities and blackguardism. But if the subject of discussion is to be the relative merits of two schools; or, worse still, the motives, candor, or veracity of the combatants, we must decline any further intercourse. In such a quarrel our readers have no interest. We have no Corporal Bullhead in our service to do the smutty part of the work, and we have no inclination to do it ourselves.

The eagerness with which this error of a paragraph is seized upon for personal invective, while all sound argument is sedulously avoided, brings with it the conviction that the "Peninsular" answers the question of fact because it *can* do so, but pays no heed to the main issue, because it *cannot* reply to the arguments we have adduced.

We had hoped to see in the "Peninsular" a calm and honest reply to our articles on the general subject of medical colleges. We have no quarrel with the school at Ann Arbor, its conductor, or its organ. But we are willing to discuss the merits of the system on which that school is founded, as compared with the system of individual enterprise.

In conclusion, we repeat that it would be lowering the dignity of our Journal to engage in such a discussion as the "Peninsular" has commenced. When it can meet us good-naturedly, and without calling hard names, we shall, so far as possible, reinstate it in our former good-will. S. B. H.

